# The role of the microbiome in endometrial carcinoma: Pathogenesis, biomarkers, and therapeutic prospects

**DOI:** 10.1111/jog.70070

**Published:** 2025-09-08

**Authors:** Wafa Abdalla, Wisam Nabeel Ibrahim, Atiyeh M. Abdallah, Maha Abdulla Al‐Asmakh, Sawsan Sudqi Said

**Affiliations:** ^1^ Department of Biomedical Sciences, College of Health Sciences, QU‐Health Qatar University Doha Qatar

**Keywords:** endometrial cancer, microbiome, tumor microenvironment

## Abstract

**Aim:**

Recent studies show that human microbiomes play a significant role in the development of endometrial carcinoma, which is a common gynecological cancer affecting women of reproductive age. This review provides an extensive analysis of how the microbiome interacts with endometrial carcinoma while focusing on its impact on disease progression and potential therapeutic advancements.

**Methods:**

Extensive literature research was conducted to examine how microbial dysbiosis affects endometrial cancer development. The research analyzed both animal model studies and clinical cohort data to assess how microbiome composition influences cancer risk and progression alongside treatment outcomes. The research explored possible microbiome interventions alongside the translational challenges they present.

**Results:**

New research findings demonstrate that microbial imbalance contributes to endometrial cancer development through chronic inflammatory processes and estrogen metabolism changes while affecting immune system behavior in the tumor microenvironment. Scientists are exploring unique microbial patterns to serve as biomarkers for detecting diseases and predicting treatment outcomes. Microbiome‐targeted strategies, including probiotics and diet changes, demonstrate the potential to enhance treatment results for individuals receiving immunotherapy and chemotherapy.

**Conclusions:**

This review examines the detailed interactions between the microbiome and endometrial carcinoma and outlines their importance in developing predictive models and innovative treatments. We explore both the obstacles faced in applying microbiome research to clinical settings and potential research paths that could speed up the integration of microbiome‐based therapies into patient healthcare.

## INTRODUCTION

Endometrial cancer is the most prevalent gynecological malignancy, arising through uncontrolled proliferation of endometrial cells to form a tumor within the uterine cavity.^1^ There are several histopathological subtypes of endometrial cancer, each with distinct clinical and molecular features.^2^ The most common histological type is endometrioid endometrial cancer, which accounts for 75%–80% of cases and includes several subtypes such as adenocarcinoma with squamous differentiation, adenoacanthoma, adenosquamous carcinoma, secretory carcinoma, ciliated carcinoma, and villoglandular adenocarcinoma. These tumors are graded from 1 (low grade) to 3 (high grade) based on their degree of differentiation. Early stage endometrioid carcinomas generally have a favorable prognosis, with a 5‐year survival rate of approximately 96% in lymph node‐negative cases, decreasing to 67% in lymph node‐positive cases. In contrast, non‐endometrioid subtypes, including serous, clear cell, and carcinosarcoma, are less common but more aggressive, with serous carcinoma accounting for only 10% of cases but contributing to a disproportionately high number of deaths due to its rapid progression and metastatic potential.^3^, ^4^

Endometrial cancers are classified into two major pathological groups: type I and type II tumors. Type I tumors, which are typically estrogen‐dependent, consist mainly of low‐grade endometrioid carcinomas (grades 1–2) and are associated with favorable outcomes. Type II tumors, on the other hand, are estrogen‐independent, high‐grade (grade 3) non‐endometrioid carcinomas, characterized by aggressive clinical behavior and poorer prognosis.^3^

Endometrial carcinoma is being redefined beyond the conventional type I/II classification, increasingly focusing on its molecular characteristics. According to The Cancer Genome Atlas (TCGA) and subsequent classifiers like ProMisE, we now recognize four primary molecular subtypes: (i) POLE‐ultramutated tumors, which are linked to an excellent prognosis; (ii) microsatellite instability‐high (MSI‐H) tumors, associated with intermediate outcomes; (iii) copy‐number low (endometrioid‐like) tumors, which exhibit a variable prognosis; and (iv) copy‐number high/p53‐abnormal (serous‐like) tumors, connected to the poorest outcomes. This molecular stratification offers a more reliable prediction of prognosis than the traditional Bokhman model and is increasingly influencing treatment decisions. Additionally, emerging evidence indicates that the tumor‐associated microbiome may differ among molecular subtypes, suggesting an important relationship that deserves further investigation.^5^

Endometrial cancer is relatively common and its global incidence is increasing. It therefore represents a significant health burden on women. In 2022, there were an estimated 420 242 new cases worldwide, resulting in 97 704 deaths, representing a mortality rate of nearly 25%.^6^ The disease predominantly affects postmenopausal women, with the highest incidence occurring between the ages of 50 and 69 years.^7^ The rising incidence underscores the need for a comprehensive understanding of the underlying mechanisms driving its development and progression to inform detection and prevention strategies.

Anatomically, the uterus consists of three layers: the endometrium, myometrium, and perimetrium, with endometrial cancer arising from the innermost layer.^3^ The endometrium is a highly dynamic tissue formed from two distinct layers: the basalis layer, which remains relatively stable, and the functionalis layer, which undergoes cyclical shedding during menstruation. The endometrium also shows cellular heterotypia, consisting of luminal and glandular epithelial cells and stromal cells, all of which contribute to its functional and structural integrity.^8^ The luminal epithelium plays a crucial role in embryo implantation, while the glandular epithelium produces secretions that support embryonic development. Stromal cells provide structural support and mediate cyclical changes in response to hormones.^8^, ^9^

Endometrial physiology is primarily regulated by ovarian hormones, estrogen, and progesterone. During the proliferative phase of the menstrual cycle, estrogen stimulates endometrial growth and thickening. In the secretory phase, progesterone induces glandular secretions to prepare for potential implantation.^8^ However, dysregulation of these hormonal pathways can contribute to endometrial carcinogenesis. Several well‐established risk factors contribute to the development of endometrial cancer, including obesity, diabetes, hypertension, and a sedentary lifestyle. These modifiable factors are implicated in the increasing incidence of the disease, particularly in high‐income countries.^10^

There is also a growing body of evidence suggesting that the microbiome plays a crucial role in the pathogenesis of endometrial cancer. Microbial dysbiosis may influence tumor development by modulating local inflammation, estrogen metabolism, and immune responses. Understanding the interplay between the microbiome and endometrial cancer could lead to novel diagnostic and therapeutic approaches.

Currently, it remains unclear whether the impact of the microbiota is more significant than that of traditionally recognized risk factors such as hormonal imbalance, genetic mutations, and environmental exposures. However, the impact of various risk factors for endometrial carcinoma varies significantly. Obesity, unopposed estrogen exposure, and metabolic syndrome stand out as the most significant contributors. In contrast, age, family history, and certain genetic mutations play a role but to a lesser degree. It is also becoming increasingly clear that the microbiome interacts with these established risk factors.^11^ Therefore, future studies should assess whether these factors act independently, synergistically, or whether one pathway dominates the carcinogenic process.

## PATHOGENESIS OF ENDOMETRIAL CANCER

The pathogenesis of endometrial cancer is complex and multifactorial, involving genetic, hormonal, and environmental factors. Identified risk factors include obesity, diabetes, hypertension, Lynch syndrome, microbiota dysbiosis, and the use of estrogen‐only hormone replacement therapy.^12^

### Molecular pathology

**FIGURE 1:**
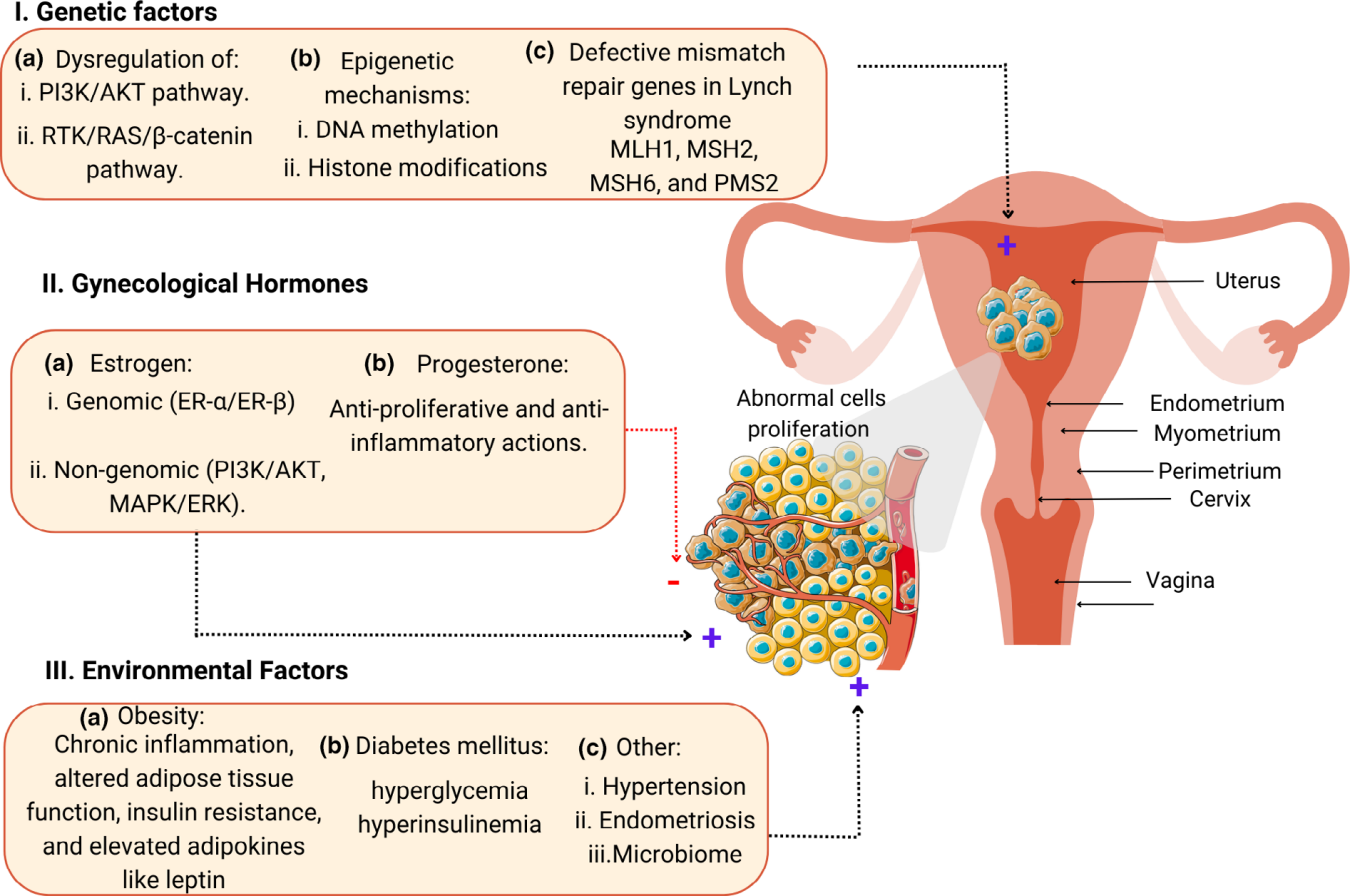
Factors contributing to endometrial cancer pathogenesis. (I) Genetic factors: (a) phosphatidylinositol 3‐kinase/protein kinase AKT (PI3K/AKT) pathway: Dysregulation causes excessive protein activation, leading to uncontrolled cancer cell proliferation. Receptor tyrosine kinase/renin‐angiotensin system/β‐catenin (RTK/RAS/β‐catenin) pathway: Mutations in associated genes drive cellular transformation and cancer progression. (b) Epigenetic mechanisms. Aberrant DNA methylation patterns, such as hypermethylation of tumor suppressor genes and hypomethylation of oncogenes, dysregulate cellular pathways related to hallmarks of cancer. Histone modifications alter chromatin accessibility, influencing gene expression and contributing to disease progression. (c) Lynch syndrome: Mutations in mismatch repair genes (e.g., *MLH1*, *MSH2*) impair genomic integrity and increase cancer risk. (II) Gynecological hormones: (a) Estrogen: promotes endometrial proliferation via genomic (estrogen receptor activation) and non‐genomic (PI3K/AKT, MAPK/ERK) mechanisms. (b) Progesterone: acts as a tumor suppressor, inducing differentiation and preventing endometrial cancer progression. (III) Environmental factors: (a) Obesity: causes chronic inflammation and promotes tumor proliferation through adipokines and cytokines. (b) Diabetes mellitus: hyperglycemia and hyperinsulinemia stimulate endometrial cell growth via the PI3K/AKT pathway. (c) Other factors: hypertension, endometriosis, and gut microbiome dysbiosis can induce inflammation and hormonal imbalances, contributing to carcinogenesis. Created with Canva.com.

Extensive research has identified key genetic and epigenetic alterations contributing to endometrial cancer development and progression. Two critical signaling pathways implicated are the phosphatidylinositol 3‐kinase (PI3K)/protein kinase AKT (PI3K/AKT) and the receptor tyrosine kinase (RTK)/renin‐angiotensin system (RAS)/β‐catenin pathways (Figure 1, I‐a). The PI3K/AKT pathway regulates cell growth, proliferation, and survival, and its dysregulation promotes excessive cancer cell proliferation.^13^, ^14^ Similarly, the RTK/RAS/β‐catenin pathway influences cell growth, differentiation, and migration, with frequent mutations in the genes encoding pathway members observed in endometrial cancer.^15^ Emerging evidence suggests that the interplay between these pathways exacerbates tumor progression, highlighting their potential as therapeutic targets.^16^, ^17^ Epigenetic modifications, such as DNA methylation and histone modifications, also play crucial roles in endometrial cancer. Aberrant DNA methylation, including hypermethylation of tumor suppressor genes and hypomethylation of oncogenes, disrupts cellular pathways controlling proliferation, apoptosis, and metastasis.^18^ Histone modifications, such as acetylation and methylation, influence chromatin accessibility and gene expression, contributing to carcinogenesis.^19^ A recent study identified increased methylation of *BHLHE22* in endometrial carcinogenesis, which was linked to a pro‐inflammatory tumor microenvironment (TME) characterized by high infiltration of CD8^+^ lymphocytes and CD68^+^ macrophages.^20^ Identifying and understanding the specific epigenetic alterations that occur in endometrial cancer could pave the way for the development of targeted therapeutic interventions, as well as the identification of novel biomarkers for early detection and disease monitoring (Figure 1, I‐b).^15^

Lynch syndrome, a hereditary condition caused by mutations in mismatch repair (MMR) genes such as *MLH1*, *MSH2*, *MSH6*, and *PMS2*, predisposes individuals to various cancers, especially colorectal and endometrial cancer. Defects in MMR genes contribute to genomic instability and tumorigenesis, making endometrial cancer the second most common malignancy in Lynch syndrome patients (Figure 1, I‐c).^21^, ^22^

### Hormonal influences

Hormonal imbalances, particularly prolonged exposure to unopposed estrogen, are central to endometrial carcinogenesis. Estrogen stimulates endometrial proliferation through genomic and non‐genomic mechanisms. The genomic pathway involves estrogen receptor (ER‐α and ER‐β) activation, leading to transcriptional regulation of target genes involved in proliferation and differentiation. The non‐genomic pathway involves rapid activation of signaling cascades such as PI3K/AKT and Mitogen‐activated protein kinase/extracellular signal‐regulated kinases (MAPK/ERK), further promoting cancer progression.^23^ Estrogen resistance, often observed in endometrial cancer, may arise due to receptor mutations and activation of alternative pathways, complicating endocrine treatment strategies (Figure 1, II‐a).^24^

Progesterone serves as a counterbalance to estrogen, exerting anti‐proliferative and anti‐inflammatory effects. Its absence or resistance is linked to endometrial hyperplasia and cancer development. Progesterone promotes cellular differentiation and suppresses endometrial proliferation, highlighting its therapeutic potential in endometrial cancer management (Figure 1, II‐b).^25^

### Environmental factors

#### Obesity

Obesity is a well‐established risk factor for endometrial cancer, primarily due to its influence on hormonal and metabolic pathways. Adipose tissue is not merely an energy storage site but an active endocrine organ that secretes adipokines such as leptin and adiponectin. Leptin promotes cell proliferation, migration, and angiogenesis through activation of Janus kinase/signal transducer and activator of transcription (JAK/STAT) and PI3K/AKT signaling, whereas adiponectin has anti‐inflammatory and anti‐proliferative effects (Figure 1, III‐a).^26^ Obesity is associated with increased levels of circulating estrogen due to enhanced aromatization of androgens in adipose tissue, leading to a state of chronic estrogen stimulation of the endometrium. Furthermore, obesity‐induced inflammation results in the production of pro‐inflammatory cytokines, such as interleukin‐6 (IL‐6), tumor necrosis factor‐alpha (TNF‐α), and C‐reactive protein (CRP), which contribute to an inflammatory TME conducive to cancer progression.^27^ Obesity‐induced changes in the TME can lead to the recruitment and activation of pro‐tumorigenic cells, such as tumor‐associated macrophages (TAMs) and myeloid‐derived suppressor cells (MDSCs), which can further enhance tumor growth and angiogenesis.^27^ The interplay between insulin resistance and chronic inflammation further exacerbates endometrial cancer risk by promoting hyperinsulinemia, which enhances endometrial cell proliferation and inhibits apoptosis via the insulin receptor and insulin‐like growth factor 1 (IGF‐1) signaling pathways.^26^ Interestingly, comorbid obesity and Lynch syndrome can synergize, further exacerbating the risk of endometrial cancer development and progression (Figure 1, III‐a).^28^

#### Diabetes mellitus

Diabetes mellitus, particularly type 2 diabetes, is associated with an increased risk of endometrial cancer through mechanisms such as insulin resistance and hyperinsulinemia. Insulin and IGF‐1 can activate the PI3K/AKT/mTOR (Mammalian Target of Rapamycin or Mechanistic Target of Rapamycin) pathway, promoting cell proliferation, inhibiting apoptosis, and inducing angiogenesis. Hyperglycemia leads to the production of advanced glycation end‐products (AGEs), which interact with their receptors (RAGE) to trigger oxidative stress, DNA damage, and inflammatory signaling pathways such as Nuclear Factor kappa‐light‐chain‐enhancer of activated B cells (NF‐κB) and JAK/STAT, further contributing to tumorigenesis (Figure 1, III‐b).^29^, ^30^ Additionally, hyperglycemia promotes mitochondrial dysfunction and increased reactive oxygen species (ROS) production, which can lead to genomic instability and epigenetic modifications that support cancer initiation and progression.

#### Hypertension

Hypertension has been linked to endometrial cancer, possibly due to its association with obesity and metabolic syndrome. Chronic hypertension induces endothelial dysfunction, oxidative stress, and chronic low‐grade inflammation, which may promote an environment favorable to tumor development.^31^ Additionally, the RAS has been implicated in cancer progression, with angiotensin II stimulating cell proliferation, inflammation, and angiogenesis through AT1 receptor activation. Hypertension‐associated oxidative stress can lead to increased production of free radicals and impaired nitric oxide signaling, contributing to endothelial damage and altered vascular function, which may facilitate tumor growth and metastasis.^32^

#### Endometriosis

Endometriosis, a condition characterized by the presence of endometrial‐like tissue outside the uterus, has been identified as a potential risk factor for endometrial cancer. Chronic inflammation, hormonal imbalances, and genetic alterations in patients with endometriosis may contribute to malignant transformation. Inflammatory mediators such as prostaglandins, cytokines, and chemokines create a pro‐inflammatory milieu that promotes cellular proliferation and resistance to apoptosis.^33^ Altered immune responses, characterized by dysfunctional natural killer (NK) cells and macrophages, may further contribute to immune evasion and cancer development. Additionally, oxidative stress and increased ROS production in endometriotic lesions can lead to DNA damage and genetic instability, favoring progression to malignancy.^34^, ^35^

#### Environmental exposures

Environmental factors, such as exposure to endocrine‐disrupting chemicals (EDCs), may also contribute to endometrial cancer risk. EDCs, including bisphenol A (BPA), phthalates, and polycyclic aromatic hydrocarbons (PAHs), can interfere with hormonal signaling by mimicking estrogen and disrupting endocrine homeostasis.^36^ Persistent exposure to these chemicals has been associated with increased estrogenic activity and endometrial proliferation. EDCs can also induce oxidative stress, DNA damage, and epigenetic modifications that may contribute to cancer initiation and progression.^37^

#### Gut microbiome

Recent studies have highlighted the gut microbiome as a potential contributor to endometrial cancer pathogenesis. The gut microbiome, a dynamic ecosystem of microorganisms, plays a critical role in maintaining metabolic, immune, and endocrine homeostasis, with dysbiosis—an imbalance in microbial composition—increasingly linked to carcinogenesis.^38^, ^39^ Dysbiosis may promote endometrial cancer through mechanisms involving chronic inflammation, estrogen metabolism disruption, and metabolic disturbances, all of which can create a tumor‐promoting microenvironment.^40^ A significant mechanistic insight into this association involves the disruption of gap junction intercellular communication (GJIC), which is crucial for maintaining cellular homeostasis by regulating the exchange of ions, metabolites, and signaling molecules between adjacent cells. Aberrant expression and mislocalization of connexins, the key structural proteins of gap junctions, have been documented in endometrial cancer, leading to impaired intercellular communication and enhanced tumorigenic potential.^41^ Loss of GJIC has been implicated in uncontrolled proliferation, resistance to apoptosis, and enhanced invasive capabilities of cancer cells, suggesting it plays a pivotal role in early tumorigenesis.^42^

In conclusion, the development of endometrial cancer is driven by a complex interplay between metabolic, hormonal, and microbial factors, with obesity, diabetes, hypertension, gut dysbiosis, and endometriosis serving as critical contributors or mediators. Addressing these risk factors through lifestyle interventions, targeted therapies, and deeper exploration of their associated molecular mechanisms may offer promising strategies for prevention and management.

## THE TUMOR MICROENVIRONMENT OF ENDOMETRIAL CANCER

The TME plays a crucial role in the development and progression of endometrial cancer. The interaction between cancer cells and their surrounding microenvironment regulates various aspects of tumor progression in different cancer types. The TME in endometrial cancer contains a diverse array of immune cells, stromal components, and extracellular matrix (ECM) components, forming a dynamic ecosystem that can either support or inhibit tumor growth.^43^ Key cellular components of the TME include cancer‐associated fibroblasts (CAFs), immune cells, and endothelial cells, which collectively influence cancer progression through intricate signaling networks.^44^

Endometrial cancer TME is characterized by distinct biochemical and biophysical properties, such as hypoxia, acidosis, high interstitial fluid pressure, and increased ECM stiffness. These factors contribute to cancer cell metabolic reprogramming, influencing proliferation, invasion, and therapeutic resistance.^44^ Identifying the key signaling pathways and peptides synthesized by stromal cells is crucial for developing potential biomarkers of metastasis and high‐grade malignancy in endometrial cancer.

Fibroblasts, the predominant stromal cells within the TME, actively remodel the ECM by producing collagen and fibronectin, establishing a pro‐tumorigenic niche. Tumor‐associated fibroblasts secrete growth factors, including fibroblast growth factor (FGF), platelet‐derived growth factor (PDGF), and vascular endothelial growth factor (VEGF), which facilitate tumor cell proliferation, invasion, and metastasis.^45^ CAFs also play a significant role in tumor angiogenesis by promoting the formation of new blood vessels, which supply oxygen and nutrients to the tumor. VEGF, a well‐studied proangiogenic factor, is pivotal in initiating the “angiogenic switch” further enhancing tumor growth and metastasis.^46^

**FIGURE 2:**
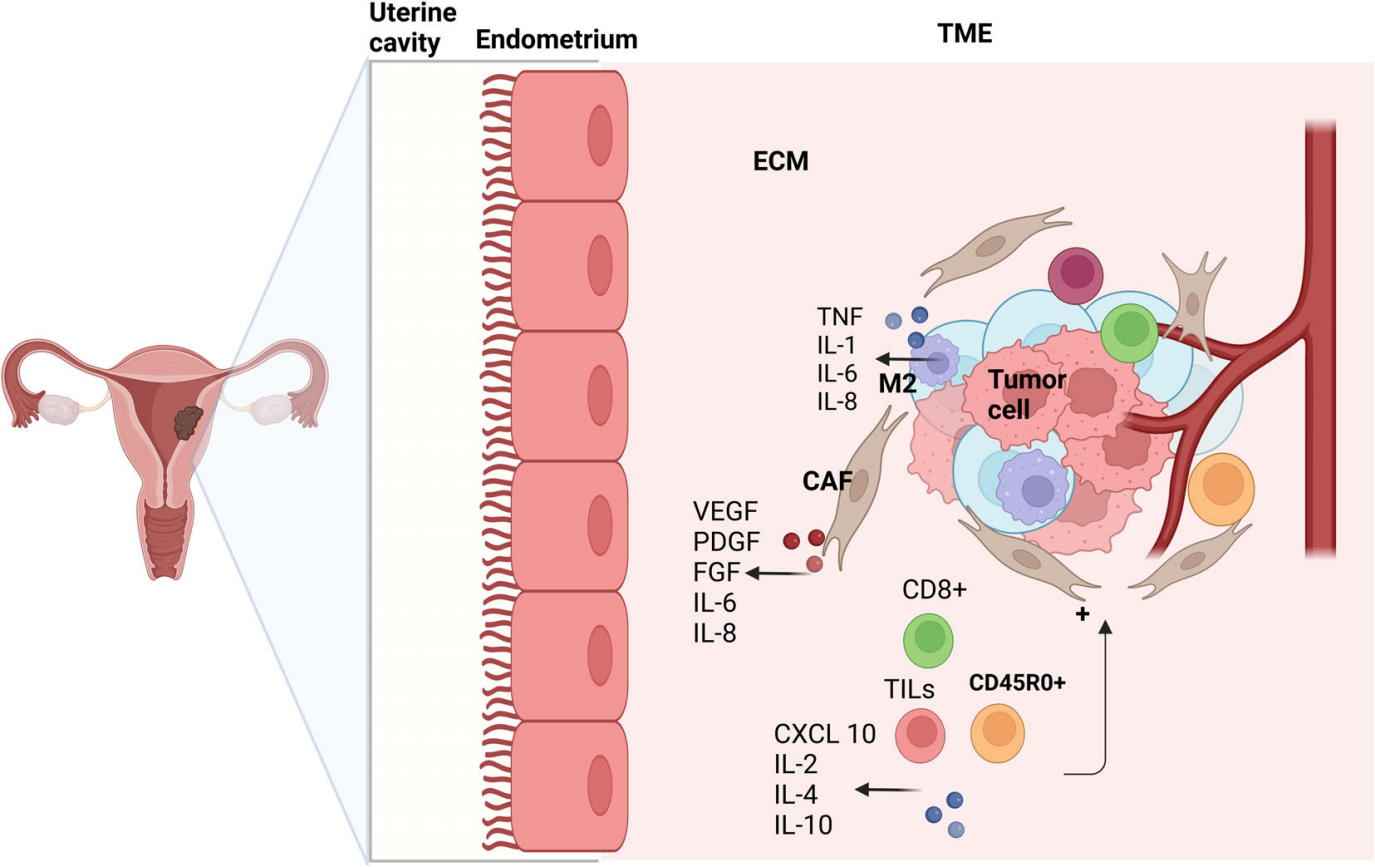
The tumor microenvironment of endometrial cancer. This figure depicts the complex tumor microenvironment (TME) of endometrial cancer, emphasizing its key cellular and molecular components. The TME comprises cancerous uterine cells surrounded by the extracellular matrix (ECM), cancer‐associated fibroblasts (CAFs), immune cells—including tumor‐infiltrating lymphocytes (TILs) such as CD8^+^ T cells, CD45R0^+^ T cells, and tumor‐associated macrophages (M2)—as well as endothelial cells and adipocytes. CAFs remodel the ECM and secrete factors like fibroblast growth factor (FGF), platelet‐derived growth factor (PDGF), and vascular endothelial growth factor (VEGF), promoting tumor proliferation, invasion, and angiogenesis. Immune cells play dual roles; CD8^+^ and CD45R0^+^ T cells enhance anti‐tumor responses, whereas tumor‐associated macrophages and regulatory T cells contribute to immune evasion and tumor progression. VEGF‐driven angiogenesis supports tumor growth by supplying nutrients and oxygen. The TME is characterized by hypoxia, acidosis, and increased ECM stiffness, which influence cancer metabolism and therapy resistance. Additionally, interleukins (interleukin [IL‐1], IL‐2, IL‐4, IL‐6, IL‐8, IL‐10) and tumor necrosis factor (TNF), secreted by various cells, modulate the TME, promoting either tumor progression or immune suppression depending on the context. Created with BioRender.com.

The immune landscape of the endometrial cancer TME consists of various immune cell populations, including tumor‐infiltrating lymphocytes (TILs) and CD8^+^ T cells. TILs, which include infiltrates of T cells, B cells, and NK cells, are critical in mediating anti‐tumor immune responses. Among them, CD45RO^+^ memory T cells are of particular interest, as their presence has been linked to improved overall survival in type II endometrial cancer.^47^ These cells mount rapid responses upon antigen re‐exposure and secrete chemokines such as CXCL10, which attract additional anti‐tumor immune cells, including NK cells, to the tumor site.^48^ However, tumor‐infiltrating regulatory T cells (Tregs), recruited via chemokines such as CCL22, suppress the anti‐tumor immune response, creating an immunosuppressive microenvironment.^49^ The prognostic significance of intraepithelial CD8^+^ T cells, particularly those expressing CD103, highlights their role in tumor control and patient outcomes.^50^, ^51^ Macrophages are another critical immune component within the TME, exhibiting functional plasticity. TAMs, often polarized toward an M2‐like (immunosuppressive, pro‐carcinogenic) phenotype, promote cancer progression by secreting proangiogenic factors such as VEGF and enhancing tumor cell invasion and metastasis.^27^ TAMs contribute to immune evasion through the secretion of immunosuppressive cytokines, including IL‐10 and transforming growth factor‐beta (TGF‐β), further suppressing anti‐tumor immunity. Moreover, cytokines such as IL‐1, IL‐6, and IL‐8 secreted by TAMs and fibroblasts enhance tumor growth, angiogenesis, and metastasis.^52^ Conversely, certain cytokines, such as IL‐2 and IL‐12 secreted by lymphocytes, can stimulate immune responses against tumor cells. TNF‐α, primarily produced by macrophages, exhibits dual roles in cancer, promoting tumor progression via angiogenesis and invasion while also exerting anti‐tumor effects through immune activation.^53^, ^54^ The immune checkpoint pathways, particularly the PD‐1/PD‐L1 axis, are key mechanisms through which endometrial cancer cells evade immune detection. Targeting these pathways has led to the development of immune checkpoint inhibitors (ICIs), which have shown promise in specific molecular subtypes of endometrial cancer.^55^ The endometrial cancer microenvironment and its components are illustrated in Figure 2.

## THE ASSOCIATION BETWEEN MICROBIOTA AND ENDOMETRIAL CANCER

Approximately half of women diagnosed with endometrial cancer have well‐established risk factors such as obesity, estrogen exposure, and/or advanced age. However, the remaining cases occur in women under 40 years of age, with a healthy weight and no history of estrogen treatment, highlighting the complexity and heterogeneity of this disease.^56^ This variability has sparked significant research interest in exploring microbiome profile differences in healthy and malignant endometrial tissues.^40^ Advances in next‐generation sequencing technologies have enabled a deep analysis of the endometrial microbiome, facilitating the discovery of associations between microbial composition and cancer status.

It is well established that the majority of the female genital tract microbiota resides in the lower reproductive tract, primarily the cervicovaginal region. An imbalance in this microbiota, such as a reduction in *Lactobacillus* spp. and an overgrowth of anaerobic bacteria, including *Gardnerella* spp., *Megasphaera* spp., *Sneathia* spp., and *Prevotella* spp., can lead to bacterial vaginosis (BV), a condition associated with various gynecological disorders. Notably, while lower genital tract cancers are linked to human papillomavirus (HPV) infections, HPV does not appear to play a significant role in malignancies of the upper genital tract. Several studies have explored the interplay between vaginal and endometrial microbiota in the context of endometrial cancer. Findings suggest that the vaginal microbiome in women with endometrial cancer is enriched for anaerobic bacteria such as *Porphyromonas* spp., *Prevotella* spp., *Fannyhessea* spp., and *Pseudomonas* spp. In contrast, microbial diversity in the fallopian tubes and ovaries appears to be lower than in endometrial tissue, suggesting a distinct microbial composition along the reproductive tract.^57^ This growing body of evidence underscores the potential role of microbial dysbiosis in endometrial carcinogenesis and highlights the need for further research to identify microbiome‐based diagnostic and therapeutic strategies.

### Dysbiotic gut microbiota and endometrial cancer

**FIGURE 3:**
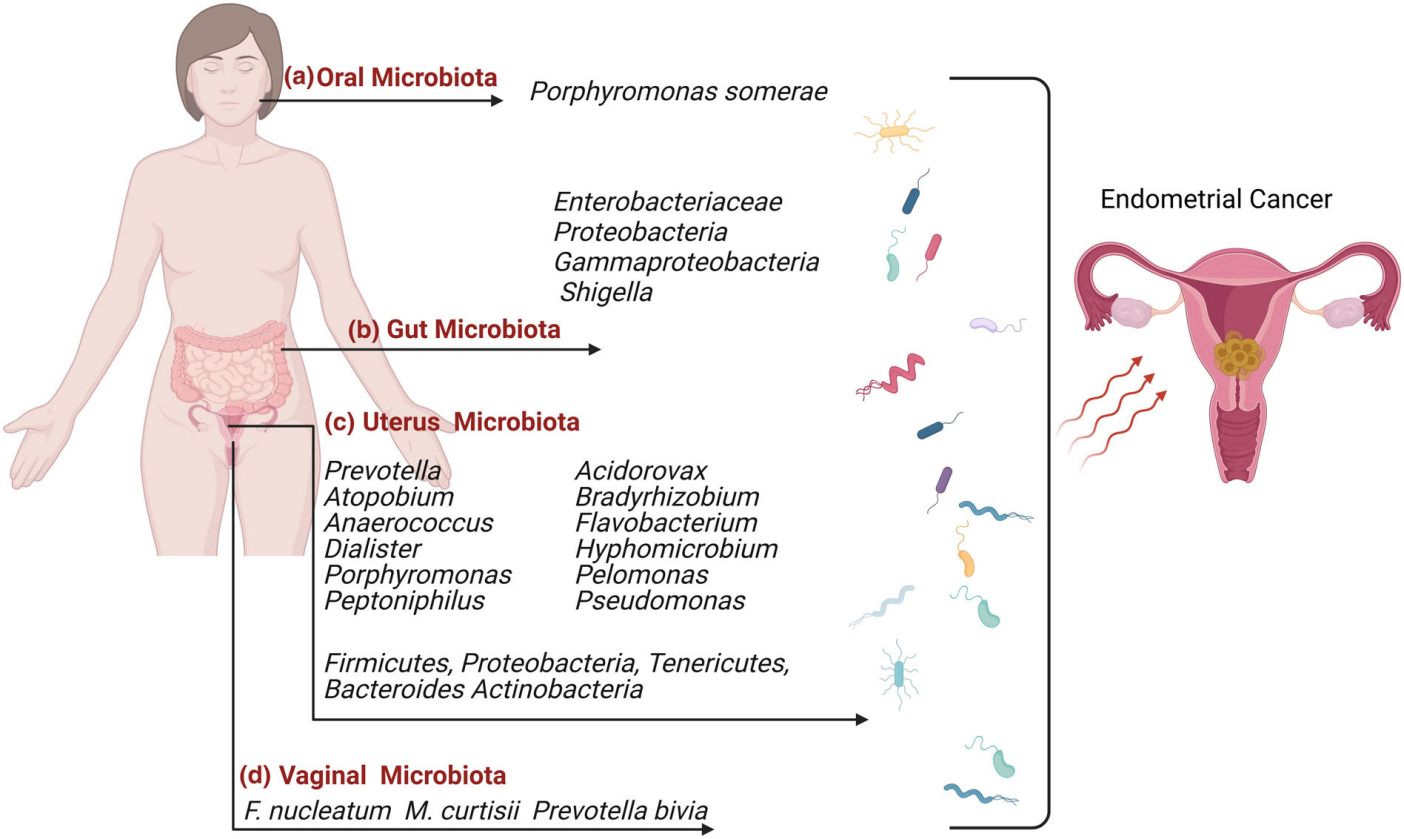
The oral cavity, gastrointestinal tract, vagina, and uterus microbiomes are associated with endometrial cancer. (a) The prevalence of *Porphyromonas somerae* has recently been recognized in the uterine environment of patients diagnosed with endometrial cancer. (b) Significantly greater numbers of *Enterobacteriaceae*, *Proteobacteria*, *Shigella*, and *Gammaproteobacteria* have been observed in the gastrointestinal microbiota of women with endometrial cancer. (c) The uterine microbiota in patients with endometrial cancer is enriched for *Prevotella*, *Atopobium*, *Anaerococcus*, *Dialister*, *Porphyromonas*, *Acidorovax*, *Bradyrhizobium*, *Flavobacterium*, *Hyphomicrobium*, *Pelomonas*, *Pseudomonas*, Firmicutes, Proteobacteria, Tenericutes, *Bacteroides*, Actinobacteria, and *Peptoniphilus*. (d) The diversity of vaginal microbiota is also variably affected in women with endometrial cancer compared with their healthy counterparts, with *Fusobacterium nucleatum*, *Mobiluncus curtisii*, and *Prevotella bivia* prevalent in those with the disease. These alterations and the translocation of microbiota may explain the influence of oral microbiota on the vaginal microbiota. Additionally, the transmission of microbiota from the oral cavity to the gastrointestinal tract to the vagina is more common in women with endometrial cancer than in healthy individuals. Created with BioRender.com.

The relationship between the gut and endometrial microbiota is still under investigation. The gut microbiota may influence circulating estrogen levels, potentially contributing to the development of estrogen‐dependent endometrial cancer.^57^, ^58^ A recent study reported significantly lower alpha diversity in the gut microbiota of patients with endometrial cancer than in healthy individuals. The gut microbiome of patients with endometrial cancer was enriched for *Enterobacteriaceae*, *Proteobacteria*, *Gammaproteobacteria*, and *Shigella*, while *Firmicutes* levels were significantly reduced (Figure 3b).^59^ The potential role of the gut microbiota in endometrial cancer pathogenesis has prompted researchers to explore factors influencing microbial composition, aiming to optimize microbiota diversity for therapeutic applications in cancer prevention and treatment.^59^

### Oral‐gut microbiota and endometrial cancer

The oral microbiota reflects systemic health and has been linked to various cancers, including pancreatic, colorectal, gastric, and endometrial cancers. For example, specific bacterial species such as *Fusobacteria* and *Leptotrichia* are associated with reduced pancreatic cancer risk, while *Porphyromonas gingivalis* and *Aggregatibacter actinomycetemcomitans* are associated with greater risk.^60^ The oral microbiome influences clinical outcomes and treatment responses, with alpha diversity impacting radiotherapy outcomes for rectal and colon cancers.^60^ Whole transcriptome sequencing has identified *Fusobacterium* in the saliva of colorectal cancer patients, with its prevalence associating with tumor subtype, immune responses, and gene expression. A high abundance of *Fusobacterium* is linked to poor survival in CMS4 colorectal cancer subtypes.^61^

Recent studies have detected *Porphyromonas somerae* in uterine tissue, suggesting oral‐uterine translocation. This bacterium produces succinic acid, which may disrupt normal uterine function and contribute to carcinogenesis.^60^

### Vaginal microbiota and endometrial cancer

The vaginal microbiota plays a critical role in reproductive health by metabolizing nutrients, interacting with other microbes, inhibiting pathogens, promoting hormonal balance, and modulating immune responses. Recent studies suggest that vaginal microbiome diversity influences endometrial cancer risk.^62^ A healthy vaginal microbiota predominantly consists of lactic acid‐producing bacteria, while patients with endometrial cancer exhibit a higher abundance of *Fusobacterium nucleatum* and *Mobiluncus curtisii*. In contrast, healthy individuals harbor *Lactobacillus crispatus*, *Lactobacillus iners*, *Lactobacillus gasseri*, and *Gardnerella vaginalis*.^63^, ^64^ Vaginal microbiota composition correlates with endometrial cancer histology and grade. *F. nucleatum* is prevalent in both high‐grade and low‐grade endometrial cancer, whereas *Prevotella bivia* and *F. nucleatum* are more common in high‐grade cases (Figure 3d). These findings suggest the potential of vaginal microbiota composition as biomarkers for endometrial cancer prediction and prognostication.^65^

### Endometrial (uterine) microbiota and endometrial cancer

The uterine microbiota, though quantitatively lower than the vaginal microbiota, exhibits greater diversity. Accurate endometrial microbiota analysis requires minimizing contamination, best achieved via surgical excision of endometrial tissue.^66^ The endometrial microbiota differs between endometrial cancer and benign cases, with endometrial cancer tissue showing greater diversity and enrichment in *Prevotella*, *Atopobium*, *Anaerococcus*, *Dialister*, *Porphyromonas*, and *Peptoniphilus* compared with peri‐cancer endometriotic tissue (Figure 3c).^40^, ^67^ Ethnic disparities in microbiota composition have also been observed, with increased microbial diversity in Black endometrial cancerendometrial cancer patients than in White patients.^68^ 16S rRNA sequencing (V3‐V5 and V4 regions) has identified *Acinetobacter*, *Actinomyces*, *Escherichia‐Shigella*, and *Rhodococcus* as unique to endometrial cancer samples, with distinct regional distribution within tumors.^66^ Further studies revealed significant depletion of *L. crispatus* in patients with endometrial cancer, with enrichment of *Anaerococcus*, *Porphyromonas*, *Prevotella*, and *Fusobacterium*.^68^, ^69^, ^70^, ^71^

*Porphyromonas* spp., particularly *P. somerae*, are implicated in estrogen upregulation via β‐glucuronidase activity, potentially influencing endometrial cancer progression.^72^ Intratumoral microbiota in endometrial cancer share similarities with cervical and ovarian cancers; however, distinct bacterial species such as *Enterococcus faecalis* (endometrial cancer), *Haemophilus parainfluenzae* (cervical cancer), and *Pleomonas* spp. (ovarian cancer) have been identified.^73^ Studies have identified over 17 bacterial taxa, including Firmicutes, Proteobacteria, and Bacteroidetes, that are more prevalent in normal endometrial tissue than in endometrial cancer. These bacteria may influence metastasis and cell adhesion through interactions with tumor cell glycosylation pathways (Figure 3c).^74^ Interestingly, these microorganisms stimulate metastasis and cell adhesion by interacting with the conversion of *N*‐acetyl‐beta‐glucosaminyl to 6‐sulfosialyl Lewis X epitope, which is mainly expressed on tumor cells to enhance adhesion to endothelial cells.^74^ The presence of specific bacteria such as *Stenotrophomonas*, *Delftia*, and *Serratia* has been associated with endometrial hyperplasia, suggesting potential early diagnostic markers for endometrial cancer progression.^75^

## MECHANISMS BY WHICH MICROBIOTA INFLUENCE ENDOMETRIAL CANCER DEVELOPMENT

Globally, microbiota are implicated in 16%–18% of malignancies. Microbial communities influence carcinogenesis through various mechanisms,^39^ including the production of genotoxins that directly damage host DNA or the integration of oncogenes, ultimately modulating cancer progression.^76^

### Metabolites produced by the gut microbiota

**FIGURE 4:**
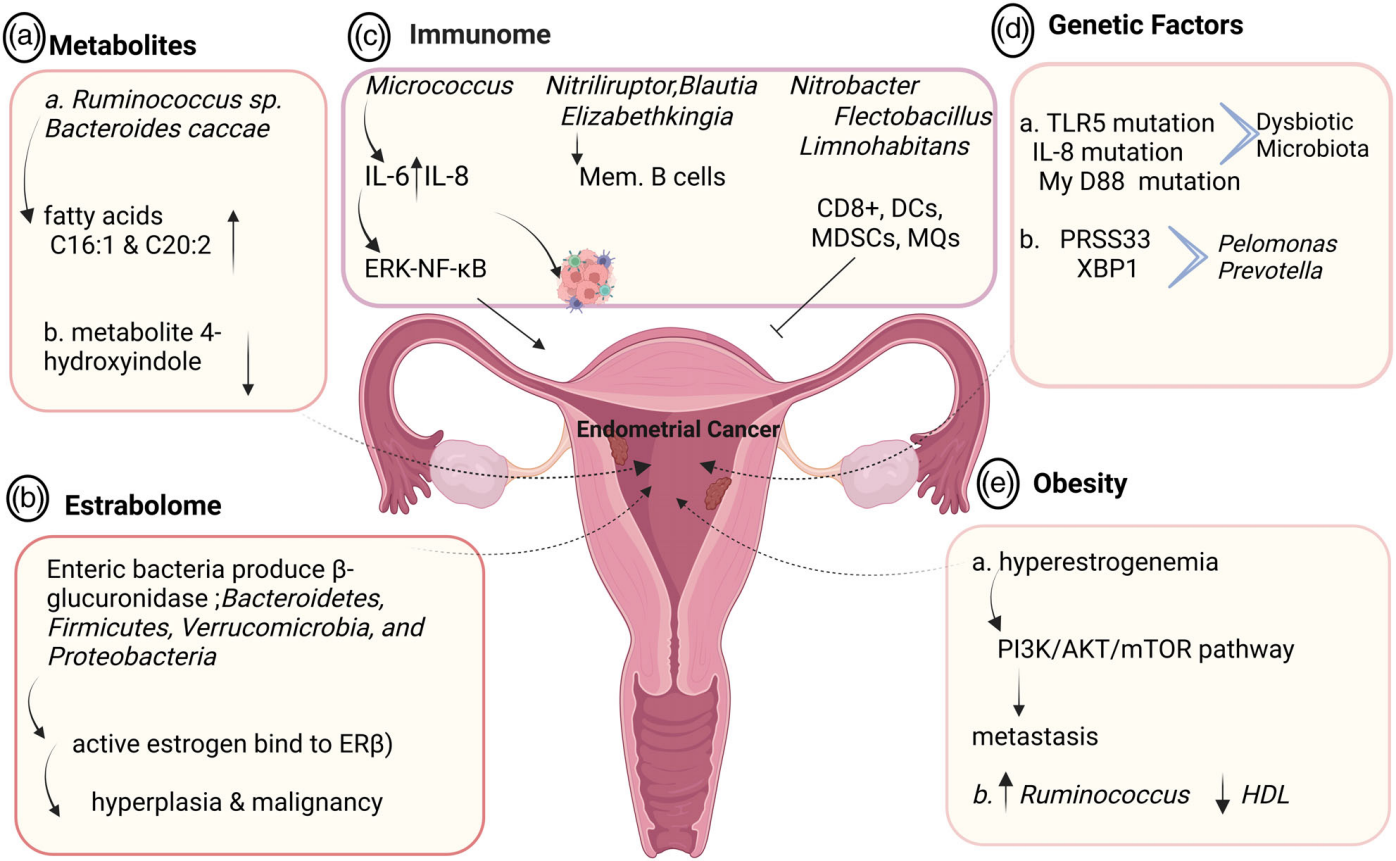
Microbiome‐endometrial cancer interactions. The underlying mechanisms of the microbiome's involvement in endometrial cancer. (a) Metabolites from specific microbiomes, such as *Ruminococcus* sp. and *Bacteroides caccae*, are elevated in the bloodstream of patients with endometrial cancer. In contrast, fatty acids like C16:1 and C20:2, along with the gut‐derived metabolite 4‐hydroxyindole, are lower in individuals with endometriosis. (b) The estrobolome consists of bacteria that produce the enzyme β‐glucuronidase, which activates endogenous estrogen, leading to hyperplasia and potential cancer. (c) Immune‐axis *Micrococcus*, found in endometrial cancer patients, is associated with increased interleukin (IL)‐6 and IL‐8 levels through ERK‐NFκB signaling, promoting endometrial tissue growth. Other bacteria like *Nitriliruptor*, *Blautia*, and *Elizabethkingia* are linked to higher memory B cell levels. Meanwhile, low‐risk endometrial cancer patients show a predominance of *Nitrobacter*, *Flectobacillus*, *Limnohabitans*, and *Cyclobacterium*, correlating with increased activated B cells, CD8^+^ T cells, and various immune cells. (d) Genetically, dysbiotic microbiota are associated with mutations in IL‐5, IL‐8, and MyD88, which affect immune cell infiltration into tumors. *Pelomonas and Prevotella* are linked to changes in PRSS33 and XBP1 in patients with endometrial cancer. (e) Obesity plays a critical role, with about 90% of type I endometrial cancer cases occurring in women with obesity. Hyperestrogenemia leads to endometrial hyperplasia via the phosphatidylinositol 3‐kinase (PI3K)/protein kinase AKT (PI3K/AKT)/mTOR pathway. Dysbiotic microbiota, especially *Ruminococcus*, negatively affect high‐density lipoprotein (HDL) levels and positively impact triglyceride levels, illustrating the microbiome's contribution to endometrial cancer and its therapeutic implications. Created with BioRender.com.

A recent comprehensive study analyzed the abundance of specific bacteria and metabolic markers in patients with endometrial carcinoma compared with healthy controls. The study identified that microbial species such as *Ruminococcus* sp. N15 MGS‐57 and *Bacteroides caccae* were significantly enriched in patients with endometrial carcinoma, correlating with elevated levels of fatty acids C16:1 and C20:2. The predominance of *Ruminococcus* sp. N15 MGS‐57 negatively correlated with the biosynthesis of betalain and indole alkaloids. Notably, mass spectrometry revealed the accumulation of fatty acid C16:1 within cancerous tissues, mirroring its systemic levels in the bloodstream. Experimental studies using Ishikawa and HEC‐1A cell lines demonstrated that exposure to fatty acids such as C16:1, C20:2, and C18:1 promoted cellular proliferation, implicating their role in tumor progression (Figure 4).^76^ Triglyceride and high‐density lipoprotein (HDL) levels in patients with endometrial carcinoma were significantly associated with *Ruminococcus* sp. N15 MGS‐57. Furthermore, obesity‐related metabolic syndrome, characterized by a dysbiotic gut microbiota and increased fatty acid levels, emerged as a predictor of cancer progression and survival. A body mass index (BMI) exceeding 30 kg/m^2^ is associated with a 4.5‐fold increased risk of endometrial carcinoma compared with non‐obese women, further underscoring the microbiota‐metabolic axis in cancer pathogenesis.^76^ The 4‐hydroxyindole metabolite (Figure 4a), a product of bacterial origin, was found to be diminished in women diagnosed with endometriosis, correlated with reduced pain, and may inhibit the progression of endometriosis.^77^

### Estrobolome

The gut microbiome influences estrogen metabolism via the estrobolome, a collection of bacterial genes involved in estrogen regulation. While estrogen governs gut microbiota composition, microbiota reciprocally influence systemic estrogen levels. Estrogen undergoes hepatic conjugation with glucuronic acid to facilitate its excretion. However, microbial species such as *Bacteroides fragilis*, *Escherichia coli*, and *Streptococcus agalactiae* produce β‐glucuronidase, which hydrolyzes conjugated estrogen, restoring its active form and enabling its binding to ER‐β. This reactivation leads to increased endometrial epithelial cell proliferation, hyperplasia, and potential malignant transformation, similar to mechanisms observed in breast cancer.^12^, ^56^ The estrobolome primarily comprises bacterial phyla Bacteroidetes, Firmicutes, Verrucomicrobia, and Proteobacteria, which collectively harbor over 112 β‐glucuronidase genes, emphasizing their significant role in estrogen homeostasis and cancer risk modulation (Figure 4b).^12^

### Microbiome‐immunome cross‐talk

The endometrial microbiota orchestrates immune responses throughout the menstrual cycle, modulating immune cell activation via pattern recognition receptors (PRRs) such as toll‐like receptors (TLRs), nucleotide‐binding oligomerization domain (NOD)‐like receptors, and Dectin‐1.^78^, ^79^ This complex interplay is crucial in maintaining homeostasis and preventing malignant transformation.

Studies have established a strong association between microbiota dysbiosis and inflammatory cytokine levels, including in IL‐6, IL‐8, and IL‐17, which are implicated in endometrial carcinogenesis. Notably, IL‐6 enhances endometrial cancer cell proliferation via the ERK‐NFκB signaling pathway, while IL‐8 facilitates immune cell recruitment to the TME. Increased expression of these cytokines correlates with the presence of *Micrococcus* in uterine cancer patients, suggesting a pro‐inflammatory milieu conducive to cancer progression.^80^ Microbiota‐immune interactions influence cancer progression through alterations in immune cell infiltration. High‐risk microbial profiles (*Nitriliruptor*, *Blautia*, and *Elizabethkingia*) are linked to the presence of memory B cells, while low‐risk profiles (*Nitrobacter*, *Flectobacillus*, and *Limnohabitans*) show increased proportions of activated B cells, dendritic cells (DCs), macrophage cells (MQs) and CD8^+^ T cells, highlighting their potential prognostic value in endometrial cancer (Figure 4c).^81^, ^82^, ^83^

### Genetic factors contributing to gut microbiota dysbiosis and endometrial cancer

Genetic predispositions may exacerbate imbalances in the gut microbiota, promoting endometrial cancer development. A bioinformatics study of TCGA data identified 13 genetic mutations associated with microbiota dysbiosis in patients with endometrial cancer. Among these, *TLR5* mutations were most prevalent (7.7%), implicating it in immune modulation and tumorigenesis. Similarly, mutations in *MYD88* and *NLRP6* were linked to altered immune cell infiltration within the TME, influencing Tregs, monocytes, and eosinophils.^84^ Additionally, mutations in *MYD88* (myeloid differentiation primary response 88) were the third most common mutation in uterine corpus endometrial cancer (UCEC) compared with 30 other cancer types. Subsequent work has demonstrated that mutations linked to gut microbiota dysbiosis play a critical role in regulating immune cell invasion within the TME of endometrial cancer, such as mutations in *NLR6* (NLR family, pyrin domain containing 6), which regulates the invasion of immune cells into the TME of endometrial cancer (Tregs, monocytes, eosinophils, follicular helper T cells, and gamma delta T cells). Notably, TLR5 is essential for regulating the invasion of M1‐like (pro‐inflammatory) macrophages and effectively activating a range of immune cells, such as CD8^+^ T cells, activated and resting CD4^+^ memory T cells, follicular helper T cells, gamma delta T cells, and myeloid dendritic cells. Its role in these processes is vital for a robust immune response.^84^

Specific microbiota, such as *Pelomonas* and *Prevotella*, correlate with increased fibrin degradation products (FDPs) and D‐dimer levels in patients with endometrial cancer, with *PRSS33* and *XBP1* significantly upregulated, promoting fibrinolysis and cancer progression (Figure 4d).^85^

### Obesity‐microbiome cross‐talk

Obesity is a major risk factor for endometrial carcinoma, particularly estrogen‐dependent type I cancers. Adipocytes contribute to cancer development through hyperinsulinemia, chronic inflammation, and hormonal dysregulation. Elevated estrogen levels derived from visceral adipose tissue stimulate the PI3K/AKT/mTOR pathway, which governs cell proliferation, survival, and metastasis. Conversely, mutations in the tumor suppressor gene *PTEN* result in unchecked PI3K/AKT/mTOR activation, promoting oncogenesis.^86^, ^87^ Obesity‐associated inflammation, characterized by increased CRP, IL‐6, leptin, and TNF‐α, further exacerbates endometrial cancer progression by enhancing cancer cell invasion and inhibiting apoptosis. Elevated CRP levels associate with poor survival outcomes, particularly in Asian populations (Figure 4e).^88^, ^89^ Additionally, gut microbiota composition analysis in patients with obesity and endometrial cancer demonstrates increased *Ruminococcus* sp. N15.MGS‐5, which correlates with dysregulated lipid metabolism and hormonal imbalances, further supporting the microbiome's role in cancer pathogenesis.^76^

In summary, the gut microbiota influences endometrial cancer through metabolic, hormonal, immune, genetic, and obesity‐related pathways. Understanding these mechanisms may provide novel therapeutic targets for personalized interventions and improved clinical outcomes.

## CLINICAL APPLICATIONS OF MICROBIOME RESEARCH IN ENDOMETRIAL CANCER

Hysterectomy is often the primary means to address endometrial pathologies, but, despite its efficacy, the procedure carries risks and it has adverse implications for female fertility, particularly among those of reproductive age. Consequently, the development of diagnostic modalities for the early detection of endometrial cancer may help increase the proportion of patients eligible for uterus‐sparing procedures or alternative therapeutic approaches that exploit hormonal or microbiome interventions.^90^

### The microbiome as a therapeutic target in patients receiving chemotherapy and immunotherapy

Radiotherapy and chemotherapy are the cornerstone of treatment for many cancers; however, up to 80% of patients experience side effects, not least gastrointestinal complications such as intestinal pain and diarrhea. The interaction between chemotherapy and microbiomes significantly influences treatment outcomes, either enhancing efficacy or causing negative effects. Doxorubicin, a widely used cytotoxic chemotherapy, may not be tolerated due to its toxicity, and it damages the intestinal endothelium regardless of the gut microbiota. Doxorubicin inhibits both cancer cell proliferation and bacterial growth.^12^, ^91^ Surprisingly, specific microbiomes have been shown to detoxify the cumulative effects of doxorubicin. Approximately 50% of administered doxorubicin is excreted through feces, interacting with the gut microbiota. *Raoultella planticola* plays a crucial role in inactivating doxorubicin through glycosylation, effectively reducing its toxicity and side effects.^91^ Understanding the contribution of the gut microbiota to cytotoxic pharmacodynamics may offer insights into optimizing chemotherapy regimens and mitigating adverse reactions.

### Modulation of immune checkpoint inhibitors by microbiota

Immunotherapy, particularly ICIs, is now the “fourth pillar” of cancer care. However, most ICIs are still in the preclinical stage, with limited studies on immune checkpoint expression in endometriotic tissues. Recent research has identified higher mRNA expression of V‐domain immunoglobulin suppressor of T cell activation (*VISTA*) in endometriotic lesions compared with normal endometrial tissue.^92^ VISTA functions by inhibiting T cells under acidic pH conditions typical of the TME.^93^ Notably, VISTA expression is positively correlated with ER levels, suggesting a hormonal influence on immune regulation. Ongoing investigations aim to determine how peritoneal microbiota diversity and their metabolites influence VISTA expression. Studies have reported an inverse correlation between the presence of *Escherichia* and *Shigella* in peritoneal fluid and the severity of endometriosis, providing potential insights into disease pathophysiology.

Furthermore, microbial metabolites such as 2‐*n*‐propylthiazolidine‐4‐carboxylic acid and LTD4‐d5 (cysteinyl leukotriene) have been found to enhance CD8^+^ T cell infiltration into the TME, potentially modulating immune checkpoint pathways in endometriotic tissue.^94^ The influence of the gut microbiota on immunotherapy responses was recently investigated in the phase II PRIMMO clinical trial (NCT03192059), which involved patients with cervical and endometrial cancers receiving pembrolizumab. The presence of the *Blautia* genus from the *Lachnospiraceae* family, alongside butyrate‐producing bacterial taxa, correlated with favorable therapeutic outcomes before treatment initiation. However, after treatment, both positive and negative correlations with the *Blautia* genus were observed, with *Lachnospiraceae* species predominating in patients exhibiting progression‐free survival (PFS) of over 12 months.^95^

### Microbiome biomarkers for diagnosis and prognosis

Atypical endometrial hyperplasia, if left untreated, carries a significant risk of progression to endometrial carcinoma, emphasizing the need for effective early detection and monitoring strategies. Current diagnostic approaches primarily depend on invasive methods, such as endometrial biopsy and hysteroscopy, which, despite their diagnostic accuracy, are associated with patient discomfort and procedural risks. Given the increasing recognition of the intricate relationship between the microbiome and endometrial oncogenesis, there is growing interest in the development of non‐invasive biomarkers that leverage microbial and metabolic profiles to enhance diagnostic precision and facilitate early intervention.^90^ Emerging evidence suggests that the cervicovaginal metabolome offers valuable diagnostic potential, with cervicovaginal lavage providing a minimally invasive means to capture metabolic and microbial alterations associated with endometrial cancer. Metabolomic analyses have revealed that approximately 9% of detected metabolites originate from microbial communities, while host‐derived metabolites account for 2.6%, co‐metabolism contributes 23%, and 64% are from undefined sources. A distinctive metabolic pattern has been identified in endometrial cancer cases, characterized by significant upregulation of lipid metabolites, which constitute 65.15% of all upregulated compounds. These lipids are implicated in key oncogenic processes such as inflammation, membrane remodeling, and energy metabolism. In contrast, amino acids—critical for cellular function and metabolic homeostasis—are consistently downregulated, suggesting potential disruptions in nutrient‐sensing pathways and metabolic reprogramming associated with tumor progression.^90^ Further research is needed to elucidate the specific microbial taxa contributing to these metabolic shifts and to validate the clinical utility of microbiome‐based diagnostic panels. Integrating microbiome and metabolome profiling may offer a comprehensive, non‐invasive approach for stratifying endometrial cancer risk, distinguishing malignant from benign conditions, and informing personalized therapeutic interventions.

## FUTURE OUTLOOK AND CHALLENGES

The intricate interplay between the endometrial microbiota and epithelial cells plays a pivotal role in modulating mucosal immune responses, influencing key cellular processes such as proliferation, apoptosis, and inflammation. These interactions are fundamental in shaping the TME in both endometriosis and endometrial cancer. The microbiota can modulate gene expression pathways involved in immune surveillance and tumorigenesis, potentially altering disease progression and therapeutic responses. Despite advances in microbiome profiling, primarily through 16S rRNA sequencing and metagenomic analyses, several critical pathways governing host–microbiota interactions remain poorly characterized. A deeper understanding of the microbial composition, functional dynamics, and host interactions is essential for discovering novel diagnostic and therapeutic targets.

Future research should focus on identifying and characterizing microbe‐derived metabolites whose concentrations are altered in cancer patients, with an emphasis on those that influence the uterine microenvironment. These metabolites may act as key modulators of endometrial homeostasis by affecting immune cell infiltration, inflammation, and hormone metabolism. Furthermore, while the balance (or otherwise) of the uterine microbiota is crucial in the pathogenesis of endometrial cancer, it is equally important to consider other contributing factors such as genetic predisposition, environmental exposures, and hormonal fluctuations. Microbiota‐based interventions, including fecal microbiota transplantation (FMT), have shown promise in addressing therapeutic resistance in various cancers; however, their potential for endometrial cancer treatment remains underexplored. Ongoing clinical trials are investigating the efficacy of prebiotic compounds such as curcumin and baicalein, as well as probiotic strains, including *Bifidobacterium* and *Lactobacillus*, in modulating the gynecological TME and enhancing treatment efficacy.^38^, ^58^ These interventions have the potential to improve responses to chemotherapy and immunotherapy by modulating systemic and local immune responses, restoring microbial homeostasis, and mitigating treatment‐associated adverse effects.^56^ Additionally, significant racial disparities in uterine microbial composition have been observed between Black and White women, underscoring the need for larger, well‐defined, and ethnically diverse study cohorts to better understand these variations and minimize potential biases in microbiome‐based diagnostics and therapeutics.^68^ Understanding the spatiotemporal expression patterns of immune checkpoints across different stages of endometrial cancer is another crucial area of research, as microbiota‐immune system cross‐talk may influence the efficacy of ICIs. Investigating the impact of microbial dysbiosis on immune checkpoint pathways could provide new insights into resistance mechanisms and guide the development of personalized immunotherapeutic strategies.^96^

In conclusion, continued exploration of the microbiome's role in endometrial cancer pathophysiology holds significant promise for advancing precision medicine for this challenging disease. Integrating microbiome analysis with other molecular profiling techniques may lead to the discovery of novel biomarkers and therapeutic targets, ultimately enhancing individualized treatment strategies and improving clinical outcomes for patients with EC.

## AUTHOR CONTRIBUTIONS

**Wafa Abdalla:** Conceptualization; writing – original draft; writing – review and editing; visualization. **Wisam Nabeel Ibrahim:** Conceptualization; writing – review and editing; visualization. **Atiyeh M. Abdallah:** Conceptualization; writing – review and editing; supervision. **Maha Abdulla Al‐Asmakh:** Writing – review and editing. **Sawsan Sudqi Said:** Conceptualization; writing – original draft; writing – review and editing; visualization; supervision.

## CONFLICT OF INTEREST STATEMENT

The authors declare no conflicts of interest.

## Data Availability

Data sharing not applicable to this article as no datasets were generated or analyzed during the current study.
